# Browsing behavior exposes identities on the Web

**DOI:** 10.1038/s41598-025-19950-3

**Published:** 2025-10-15

**Authors:** Marcos Oliveira, Junran Yang, Daniel Griffiths, Denis Bonnay, Juhi Kulshrestha

**Affiliations:** 1 Computer Science, University of Exeter, Exeter, UK; 2 AI & Behaviour, Vrije Universiteit Amsterdam, Amsterdam, The Netherlands; 3 Computer Science, Aalto University, Espoo, Finland; 4 Université Paris Nanterre, Paris, France

**Keywords:** Scientific data, Information technology, Applied mathematics

## Abstract

How easy is it to uniquely identify a person based solely on their web browsing behavior? Here we show that when people navigate the Web, their online browsing traces produce fingerprints that identify them. Merely the four most visited web domains are enough to identify 95% of the individuals. These behavioral fingerprints are stable enough to enable high short-term re-identifiability: we demonstrate that we can re-identify 80% of the individuals in contiguous time slices of data. Our results thus show that what is perhaps the most basic feature of our online habits—namely, which sites we visit most often—is highly unique. Such a privacy threat persists even with limited information about individuals’ browsing behavior, reinforcing existing concerns around online privacy.

**Supplementary Information:**

The online version contains supplementary material available at 10.1038/s41598-025-19950-3.

## Introduction

In the era of ubiquitous technology, our online habits have become a goldmine for companies seeking to extract value from our data, often risking our privacy^1^. By looking at how we browse the Web, they learn about us, enabling them to build highly targeted advertising campaigns and monetize user profiles with third-party advertisers^2,3^. Such lucrative business models focus on tracking, understanding, and predicting individuals’ behavior^1^. However, despite the commercial success, the behavioral traits that enable this profitability remain elusive and quantitatively unexplored.

Bridging the gap between web browsing data and behavioral science, researchers have recently shown that users’ online behavior is highly predictable, revealing an average of 85% potential predictability^4^. Much of this predictability arises from users’ online habits. Despite the myriad options available online, individuals adhere to web routines (i.e., repeated browsing patterns or habitual website visits) that reduce the uncertainty about their behavior^4–7^. This innate human proclivity for habits extends to offline activities such as shopping and mobility, rendering them equally predictable^8–11^. Such high predictability enables businesses to tailor individual-level services and products, forming a cornerstone of the contemporary data economy.

While this predictability helps refine user experience by improving personalization and advertising relevance, it raises concerns about individuals’ privacy and autonomy^12^. By understanding and predicting users’ behavior, platforms can exploit their psychological vulnerabilities, drawing attention to a core worry of surveillance capitalism: predicting user behavior to modify it^1,12^. This paradigm touches on various facets of an individual’s life, such as health, well-being, consumption, and voting patterns^13–16^. To enable this practice, business models rely on the precise identification of users to target them effectively.

In diverse contexts such as shopping and mobility, this identification is facilitated by the uniqueness of individuals’ actions serving as fingerprints^17,18^. For instance, how individuals navigate within cities produces distinct signatures: merely four spatiotemporal points can pinpoint most users in mobile phone data sets containing time-location details^17^. Likewise, minimal data on purchasing activities can re-identify individuals within anonymized credit card records^18^. Such a distinctiveness in the way people behave allows behavioral traits to identify individuals, replacing traditional personally identifiable information (e.g., email address, phone number). *While our habits make us predictable, our uniqueness makes us identifiable.*

Previous works have explored similar uniqueness in online environments from a technical standpoint, shedding light on browser fingerprinting^19^ and security vulnerabilities^20,21^. For example, inadvertent leaks of browsing history can identify users. Specifically, listing which of the top 50 globally most-visited websites a user visited is an efficient fingerprint^20^. This strategy uniquely identifies individuals in a data set containing whether users have once visited websites from a predefined list. Likewise, browsing trajectories—long sequences of websites visited—reconstructed from cookies have been shown to be highly unique^22^. Similarly, unique preferences in movies^23^, Facebook interests^24^, and online sharing patterns^25^ can compromise anonymity within respective data sets. However, while previous works have characterized the specifics of users’ preferences among a fixed set of options, they have overlooked the intrinsic uniqueness embedded in users’ habits in the wild. The repeated, habitual nature of how people browse the Web may create stable behavioral fingerprints that can serve as identifiers.

In this work, we uncover how our browsing habits uniquely identify us. By examining the complete data of users’ browsing behavior, we unravel the identifying power of their habitual websites. These consistently visited web domains serve as a *behavioral* fingerprint. We demonstrate that by knowing just the top four most-visited domains of a user, we can unmask 95% of users in our data set. This staggering rate of identifiability highlights a vulnerability within our digital routines: our browsing habits are distinctive, rendering us uniquely identifiable. This identification precision persists even with data from only a limited set of domains (i.e., sparse tracking). Even more alarmingly, when analyzing the data across varied time frames, we can re-identify 80% of users across different time periods.

In summary, our study tackles gaps that remained unresolved in previous research:

- While prior works have shown that online behavior is predictable and driven by routines, it was unclear whether browsing habits themselves uniquely identify individuals; we show that habitual websites form distinctive behavioral fingerprints.
- The stability of such fingerprints over time and their potential for re-identification had not been demonstrated; we show that fingerprints persist across contiguous time slices, enabling re-identification of most users.
- Earlier studies examined technical fingerprints and server logs, focusing on browser and device properties rather than individuals’ behavior; we show that repeated browsing behavior yields unique and stable identifiers across demographics.
- The extent of identifiability under sparse tracking conditions had not been systematically quantified; we show that even limited data collections are sufficient to distinguish most users.

Together, these findings expose an inherent risk of de-anonymization rooted in our browsing habits and reinforce the concerns surrounding online privacy, emphasizing the urgency to protect our digital footprints.

## Results

We analyze users’ web browsing behavior by examining web tracking data from 2148 users in Germany, which contains 9,151,243 user visits to websites, including visit time and active seconds spent on the page, collectively spanning 49,918 unique domains (see Methods for details).

### From web browsing traces to behavioral fingerprints

**Fig. 1:**
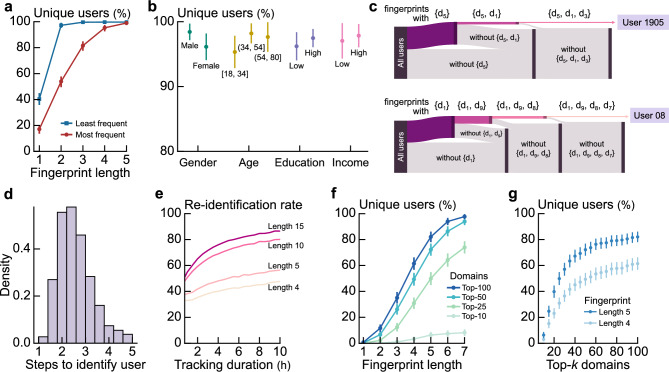
People’s habitually visited websites serve as behavioral fingerprints that distinguish them. (**a**) The percentage of users with unique most-visited domains list (i.e., fingerprint) with varying fingerprint length. Almost all users have unique four-domain fingerprints (i.e., four most visited domains). (**b**) The percentage of users with unique four-domain fingerprints grouped by age, gender, education, and income. Regardless of demographics, a four-domain fingerprint yields high uniqueness. (**c**) A schematic of a step-by-step user identification via users’ habitual websites. For each user, we randomly select a domain from their list of five most-visited domains then group all users sharing the same domain. By selecting additional domains, this process progressively refines groups, until the user is uniquely isolated. In the illustration, bar sizes represent proportional number of users. (**d**) The distribution of steps to identify a user within our data. While four domains ensure uniqueness, fewer domains are often enough for identification; by following steps in (**c**), we need an average of 2.45 steps to identify users. (**e**) The re-identification rate for different duration of data collection. The fingerprints enable re-identification of most individuals in separate time slices of data. (**f**) The percentage of unique users in scenarios of fewer tracking domains. By collecting data of a limited number of domains only, the majority of users can still be distinguished. (**g**) The percentage of unique users in scenarios of fewer tracking domains, with fixed fingerprint lengths. Collecting data from more domains yields higher uniqueness, but with decreasing returns.

To understand how online browsing behavior yields behavioral fingerprints on the Web, we analyze individuals’ most visited web domains. We demonstrate that we can uniquely identify an individual by using their list of most frequented domains. First, we describe each individual with an *n*-tuple comprising their *n* most visited domains; then, we count the number of non-duplicate *n*-tuples within our data, thereby identifying the percentage of unique users (i.e., unique fingerprints; see Fig. 1a). Our results reveal that 95% of the users have unique behavioral fingerprints when $n=4$, implying that an individual’s four most visited domains uniquely identify them.

This short-length fingerprint identifies users irrespective of gender, age, education, and income. By grouping users based on demographics and comparing their four-domain fingerprints, we find a consistent high proportion of unique fingerprints with only minimal group variations. For example, our results show a marginally higher percentage of unique male individuals at 98% compared to 96% for female individuals (Fig.1b), and similar values across age groups: 98% and 97% for the 34–54 and 55–80 brackets, with the 18–34 group slightly lower at 95%. Education and income show no meaningful differences, and we observe similar stability across other demographic attributes, including family status and presence of children (see Supplementary Note 1 and Supplementary Figure 1). We also examine how data set size influences uniqueness by subsampling users and varying fingerprint length *k*, finding that uniqueness decreases slightly as the number of users grows, but increasing *k* from 4 to 5 domains offsets this reduction (see Supplementary Note 2 and Supplementary Figure 2).

Though most users are unique in their four most-visited domains, we find that we often need fewer data points for *user identification*. To determine how many domains are needed to pinpoint a user, we examine fingerprints at the individual level. For each unique user *i*, we randomly select a domain from their fingerprint and group all unique users who have that domain in their fingerprints (see Methods). Then, we select another most-visited domain from user *i* and narrow our group to those with both domains (Fig. 1c). We repeat this step, incrementally adding domains, until we isolate user *i*. At this point, we have a set of domains which exists only within user *i*’s fingerprint. Our analysis shows that we need an average of 2.45 steps to identify a unique user within the data set (Fig. 1d). This finding indicates that although four domains guarantee uniqueness, users’ distinct online habits facilitate their identification with fewer domains.

We also assess how this uniqueness arises from behavioral regularities through data randomization, showing that identifiability hinges on individual browsing differences: making users’ browsing behavior more similar reduces uniqueness, while introducing randomness in their browsing behavior increases it (Supplementary Note 3 and Supplementary Figure 3).

We note that these behavioral fingerprints would be shorter if it were not for popular domains—more than 80% of the users share their most-visited domain with other users (Fig. 1a). These popular domains enlarge users’ fingerprints, whereas unpopular domains make them distinctive. For example, when we use the least visited domains to form the *n*-tuples, we find that most individuals have unique fingerprints when $n\ge 2$, implying that the two least visited domains of a user uniquely identify them (Fig. 1a). However, we expect considerable fluctuations in the list of the least visited domains over time, given that these may represent sporadic visits to isolated domains, which raises the question of re-identifiability based on these fingerprints.

### Using behavioral fingerprints to re-identify individuals

To assess the short-term stability of fingerprints and the potential for re-identifiability of individuals based on their fingerprint across time, we examine contiguous time slices of data and quantify re-identification rates. More precisely, we proceed in the following way: we take a certain amount of consecutive browsing by a given individual, say ten hours. We split that browsing history into two equal parts, and compare for that individual their fingerprint from the first five hours with the fingerprint from the last five hours of data. The individual is re-identifiable based on their fingerprint if and only if their fingerprint remained the same. Using ten hours of browsing data with a fingerprint of length $n = 5$, we find a 60% success rate, reaching success rate of 80% and 90% when $n = 10$ and $n = 15$, respectively (Fig. 1e).

Our results also reveal that collecting more user browsing data improves re-identification rates but with diminishing gains. For example, with a fingerprint of $n = 15$, collecting two hours of users’ browsing activity yields a re-identification rate of 65%. By extending this data collection for two hours, this re-identification raises to 76%. However, after 6 h of data collection, the gain in re-identification rate is around 2% per additional hour. Despite these diminishing returns, our findings indicate that short-duration tracking periods can be efficient for user identification. While this analysis explored the impact of tracking depth, we next examine the effect of tracking breadth.

### Using limited knowledge to identify users

We investigate the number of domains necessary for user identification, revealing that data from a limited set of domains is sufficient to identify users. First, we compile an ordered list of the most frequently visited domains by all users within our data. For each user, given a specific *k*, we only consider their visits to the Top-*k* domains in that list, effectively filtering out all other domain visits. Then, we examine the number of unique users based on fingerprints using this limited browsing data. Our analysis reveals that using data exclusively from the Top-100 domains yields unique fingerprints for 82% of users when $n=5$ (Fig. 1f). This result implies that tracking users via just 0.2% of all domains would render most users identifiable.

This uniqueness rate increases with the number of domains but with decreasing returns. We find that beyond a certain point, considering more domains only generates marginal growth in unique users. For example, while expanding the domain count from Top-25 to Top-50 boosts uniqueness rates from 50% to 75%, further expansion to Top-100 yields a relatively smaller increase to 80%. To understand this contrast, we focus on fingerprint length $n=4$ and $n=5$ while varying *k* (Fig. 1g). We observe that the percentage of unique users rises steeply with *k*, a growth that peaks at around $k=50$; after this point, the growth rate reduces, implying that additional domains contribute only incrementally to user uniqueness.

## Discussion

In a world where internet usage surpasses 5 billion people, our online habits are a valuable asset for business models relying on tracking and predicting individuals’ behavior^1,26^. However, the extent to which online behavioral traits can be exploited has remained largely unquantified. In this work, we show that online habits serve as *behavioral fingerprints* that identify individuals on the Web. Previous works have explored unique users’ preferences but have missed the uniqueness of users’ habitual websites. We demonstrate that these consistently visited domains are unique among users, revealing that individuals have distinctive habits that render them uniquely identifiable. This behavioral perspective fills a critical gap: rather than focusing on technical fingerprints, we show that routine browsing behavior itself provides a robust basis for user identification.

Our research rests on an important distinction between identifiability and re-identifiability. At a given moment in time, we say that data makes an individual *identifiable* if the portion of the data corresponding to that person is distinct from all other users. In contrast, we say that data makes an individual *re-identifiable* across two different time slices if the portion corresponding to that person is the same across both slices. In the case of browsing data, we have shown that high identifiability also goes with high-reidentifiability for adjacent time slices. While such adjacent time slices are relevant in some real-life situations (e.g., pushing ads to a targeted individual within a narrow time frame of actions), it would be interesting to look into more long term re-identifiability properties. Biological fingerprints do evolve, but only slightly: what about digital fingerprints?

At any rate, this privacy threat is critical given the growing concern among users about their digital footprints—a recent survey found that seven out of every ten people have taken actions to protect their online identity, from disabling cookies to using virtual private networks^27^. Despite such preventive actions, our work uncovers a privacy threat transcending technology, rooted in the very nature of our online browsing behavior. We tend to visit the same websites, producing consistent behavioral fingerprints that can potentially expose our identity. Such a risk is a reflection of our habits rather than technological artifacts.

Still, the online ecosystem amplifies this risk: user data collection is prevalent and concentrated in the hands of a few key players^28^. For example, Google AdSense scripts are embedded in over 51 million websites, including 20% of the top 1 million most popular sites^29^. Such a pervasive data collection enables extensive user monitoring by a single company, which is alarming given our findings that data from only a few domains is enough to identify most users. Our supplementary analysis also shows that a larger data set, or equivalently a larger number of users, slightly reduces uniqueness; however, this effect is easily offset by adding just one more domain to the fingerprint (Supplementary Note 2). Since major platforms track far more than a handful of domains, identifiability under real-world conditions is likely even higher.

In summary, our work underscores a crucial yet overlooked aspect of online privacy: habitual web visits can unwittingly compromise our digital anonymity. These browsing regularities, a natural by-product of how we browse the Web, pose a substantial privacy risk, challenging efforts to protect online identities. As the online landscape becomes more dominated by extensive data collection, our findings reinforce the urgent need to deal with privacy concerns related to all of our digital traces and not just so-called personally identifiable information, calling for innovative measures to safeguard our digital footprints.

### Limitations and future works

This work presents limitations and can be extended to understand web browsing behavior better. First, our analysis in the main text is based on a single data set. To address this, we include additional analyses in Supplementary Note 1 on two more data sets: users located in France and users located in Germany, each tracked over three months in 2020 (see Supplementary Note 4 and Supplementary Figure 4). These analyses confirm that users’ uniqueness hold across countries, longer observation periods, and different time frames. Although our data sets do not capture global diversity, they provide rare, GDPR-compliant, individual-level web browsing activity across numerous websites and across sessions, allowing us to observe consistent behavioral regularities. We find that people habitually revisit a set of websites, forming distinct digital fingerprints. While cultural factors may influence these regularities, online habits likely extend beyond cultural boundaries, similar to habits in shopping and mobility. Future research should explore the role of cultural differences in the observed regularities.

Second, we assess how the uniqueness of online habits enables user identification, relying on most-visited domains as the fingerprinting method. However, more sophisticated methods could incorporate additional browsing features, such as temporal patterns or session duration, potentially making identification more efficient. Likewise, understanding the role of specific domains (e.g., popular domains) in identification could further refine fingerprints. Such more advanced approaches offer promising directions for future research.

Third, user fingerprinting raises two relevant questions: the *static* question, which asks whether fingerprints can uniquely identify individuals at time *t*, and the *dynamic* question, which asks whether those fingerprints remain stable enough for re-identification at time $t + \Delta t$. In this work, we address the static question and the dynamic question for small values of $\Delta t$. This focus is intentional: short-term re-identification is directly relevant to the primary real-world application of behavioral tracking—targeted advertising—which typically operates on time frames of hours to days, not months or years. For example, companies such as Google, through its pervasive AdSense network embedded across millions of websites, can exploit short-term identifiability to deliver personalized ads almost immediately after visits occur. Accordingly, we make no assumption and no claim about long-term stability of behavioral fingerprints; this remains an open question for future research. Despite this limitation, our results provide critical evidence that even within short time spans, routine browsing behavior poses a substantial privacy risk.

Finally, while our work identifies privacy risks, a crucial next step will be to develop concrete mitigating strategies. Future research could explore practical solutions, such as methods that obfuscate browsing behavior or tools that help users better manage their digital footprints. Similarly, future legal and policy frameworks could address the risks of user re-identification. For instance, regulations might limit the retention of long-term tracking data to prevent the creation of extensive user trajectories that expose individuals to privacy risks.

## Methods

### Data

Our data set consists of the web activity of 2,148 German users tracked for the full month of October 2018^30^. These users were recruited through a GDPR-compliant European online panel company, provided informed consent, and received financial compensation for their participation. We use data on each user’s website visits, including the anonymized URL, its registrable domain (e.g., bbc.com from https://www.bbc.com/sport/cricket/articles/cdx5p4wy1zlo), and the date and time of the visit. To ensure privacy, the panel company removed all personally identifiable information (e.g., names, emails, addresses, passwords) before we accessed the data.

In total, the data contains 9,151,243 URL visits across 49,918 unique domains. We also examine self-reported demographic information, including gender, age, education, family status, presence of children, and income, through surveys. The sample is representative of German internet users under 65 years old in terms of gender and age^4^.

### Uniqueness, identification, and re-identification

For each user *u*, we define an *n*-tuple $m^n_u = (d^u_1, d^u_2, \cdots , d^u_n)$ as the fingerprint of *u*, where $d^u_i$ denotes the *i*th most visited web domain by this user. To analyze uniqueness given a fingerprint length *n*, we count the number of non-duplicate $m^n_u$ within our data, treating fingerprints as sets to disregard the order of elements. We use the Jackknife method^31^ to estimate the standard error of the uniqueness in our analyses (Fig. 1a, b and Fig. 1f, g).

To evaluate diversity in users’ fingerprints, we compare each unique user against all others. For a fingerprint of length *n* and for each unique user *u*, we first randomly select a domain $d^u_{r1}$ from $m^n_u$ and form a set $S(\{d^u_{r1}\})$ comprising users with $d^u_{r1}$ in their fingerprints. Next, we randomly choose another domain $d^u_{r2}$, without replacement, from $m^n_u$, and define a set $S(\{d^u_{r1}, d^u_{r2}\})$ for users having both $d^u_{r1}$ and $d^u_{r2}$ in their fingerprints. This selection process continues until $|S(\{d^u_{r1}, \cdots , d^u_{rl}\})|=1$, and the set only contains user *u*. We note that $l \le n$ and represents the number of steps (or domains) necessary to distinguish user *u* from other unique users; for each user, we find the average of *l* over 300 repetitions of this procedure (Fig. 1d).

To assess the efficacy of fingerprints in re-identifying individuals, we partition the data into two sequential time slices. First, we select a specific number of hours, denoted as $\Delta t$, to create users’ fingerprints. Each fingerprint is constructed using $\Delta t$ hours of browsing data for each user. Next, we attempt to re-identify the same users in a subsequent time slice of equal duration, the next $\Delta t$ hours, using the fingerprints generated from the first time slice. We note that same set of users who appear in both slices, and we define re-identification success deterministically: a user is considered re-identified if their fingerprint in the second slice is identical to the one from the first slice. This procedure involves no probabilistic predictions or thresholds; thus, notions such as false positives or false negatives do not apply. Instead, we compute re-identification as the proportion of users for whom this exact match occurs (akin to a hit rate). In this analysis, we use fingerprint lengths 4, 5, 10, and 15 (Fig. 1e).

## Supplementary Information

Supplementary Information.

## Data Availability

The sources of all empirical data used in our analyses are available at https://doi.org/10.5281/zenodo.4757574
