# Supplementary material for: Browsing behavior exposes identities on the Web
Source: Sci Rep. 2025 Oct 15;15:36066. doi: 10.1038/s41598-025-19950-3 (PMC12528657; doi:10.1038/s41598-025-19950-3)
Supplement: Supplementary file 1 — Supplementary Information. [file 41598_2025_19950_MOESM1_ESM.pdf]

Browsing behavior exposes identities on the Web

Supplementary Material

Marcos Oliveira, Junran Yang, Daniel Griffiths,
Denis Bonnay, and Juhi Kulshrestha

Supplementary Note 1: Demographic analysis

We analyze whether user uniqueness varies across demographic groups. We group users according to the following attributes:

- Gender: male and female.
- Age: 18–34, 34–54, and 54–80.
- Education: low education (Volks/Hauptschule, Weiterführende Schule, or no qualification) and high education (Abitur or higher).
- Children: without children and with children.
- Family status: without partner (single, divorced or widowed) and with partner (married, civil partnership, or living with partner).
- Income: low (under €1000 and €1000–2000) and high (€2000–3000, €3000–4000, and over €4000).

We compute uniqueness for each group using four-domain behavioral fingerprints, finding that uniqueness remains consistently high across all demographic categories, with only marginal differences between groups (Fig. 1).

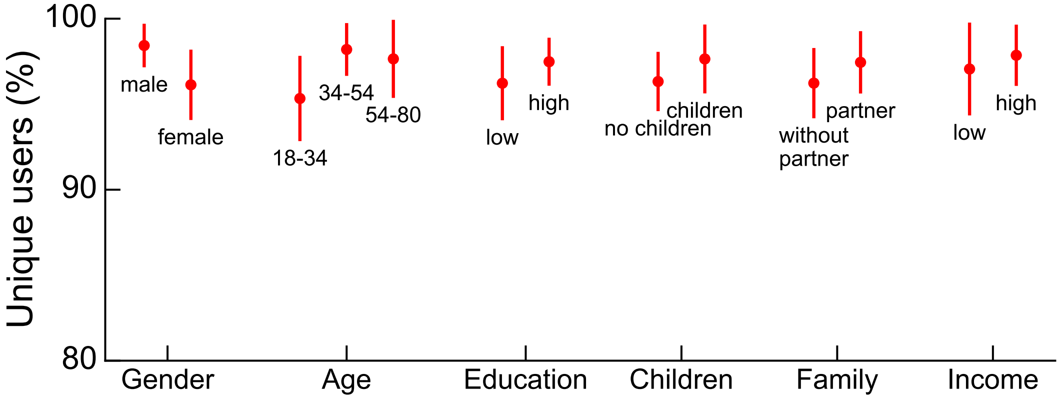

Supplementary Figure 1: Uniqueness across demographic groups: We group users by gender, age, education, family status, children, and income, and find that uniqueness remains high with no substantial differences between groups.

Supplementary Note 2: Sample size analysis

To understand the effect of number of users in the data set, we subsample users and compute uniqueness for different fingerprint lengths k. We find that uniqueness decreases slightly as sample size grows, but increasing k from 4 to 5 domains offsets this effect (see Fig. 2). This analysis is performed on the original German data set and the combined 3-months Germany–France data set.

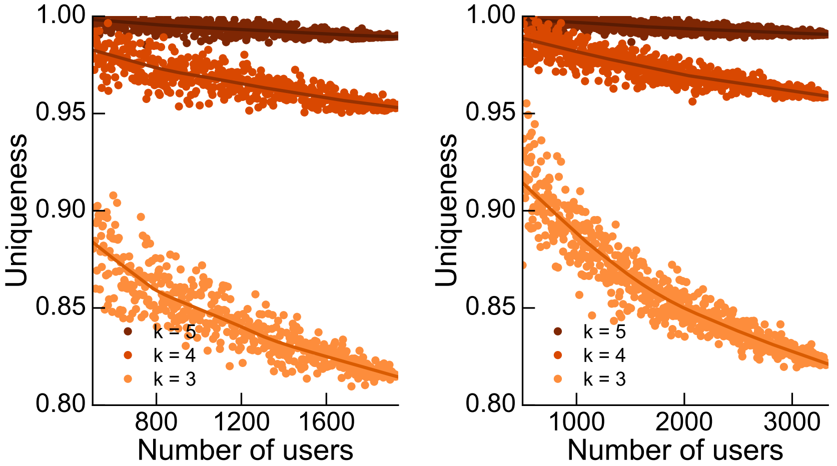

Supplementary Figure 2: Effect of sample size on uniqueness: (left) German data set (2018), (right) combined three-month German–French data set (2020). Uniqueness decreases slightly as sample size grows, but an increase in k from 4 to 5 offsets this effect.

Supplementary Note 3: Behavioral baselines using randomization models

To contextualize the role of online habits in our findings, we examine two randomization models: (1) uniform randomization, where each visited domain is replaced with a random domain from the entire data set, removing all structureas a consequence of users' browsing habits and maximizing diversity, and (2) popularity-biased randomization, where domains are replaced with another domain randomly drawn from a popularity-weighted distribution of all domains (i.e., more popular domains have a higher chance of being selected), making users converge toward popular sites and thus more similar to each other (Fig. 3). With these two models, we can contextualize the uniqueness found in data by comparing it to scenarios where habits are weakened or removed, helping us understand how

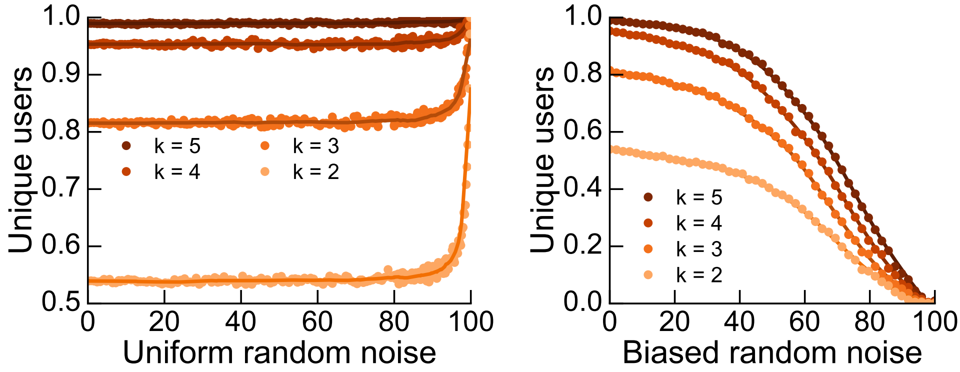

Supplementary Figure 3: Behavioral baselines: (left) Uniform randomization, (right) Popularity-biased randomization. Uniqueness increases with uniform randomization and decreases with popularity-biased randomization, demonstrating the role of browsing habits in identifiability.

much identifiability stems from consistent browsing behavior rather than random variation. To control the intensity of randomization, we vary the probability of replacing each domain in a user's fingerprint with a randomized one, from 0 (original data) to 100 (fully randomized) (see Fig. 3). As expected, uniqueness increases under pure randomization (i.e., users become highly distinct) and decreases under popularity-biased randomization (i.e., users become more alike). These results show that the identifiability observed in our findings is driven by consistent browsing habits rather than by trivial statistical artifacts.

Supplementary Note 4: Additional data sets

We assess generalizability using two additional datasets alongside the data set used in the main text (i.e., users located in Germany, tracked for one month in 2018): (a) users located in France, tracked for three months in 2020 (1,474 users, 8,731,733 URLs, and 128,270 unique domains), and (b) users located in Germany, also tracked for the same three months in 2020 (1,334 users, 7,422,352 URLs, and 136,559 unique domains). We compute uniqueness for these data sets individually and in combination, finding that high identifiability persists across countries, time periods, and tracking durations (see Fig. 4). We note that these additional data sets are collected under the same conditions as the data set used in the main text (i.e., users were recruited through a GDPR-compliant European online panel and they provided fine-grained URL-level browsing traces from their desktop devices via a tracking software installed on their machines).

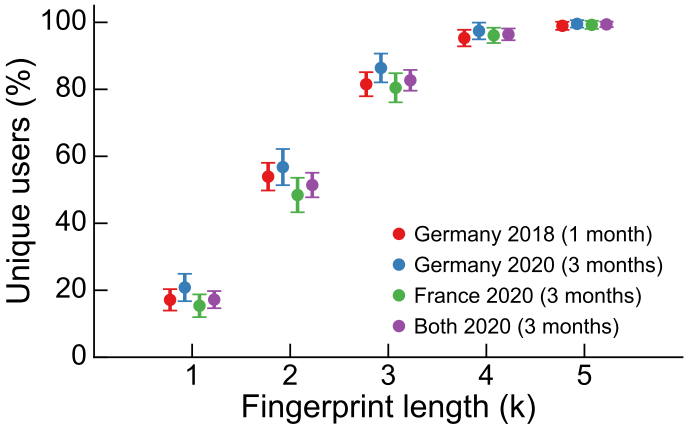

Supplementary Figure 4: Uniqueness across additional datasets: We compare user uniqueness across four scenarios: the 1-month Germany (2018) dataset, the 3-month Germany (2020) dataset, the 3-month France (2020) dataset, and the combined 3-month France + Germany dataset.
